# CONCEPTUALIZING A NEURO-ETHNOMUSICOLOGY OF AGING

**DOI:** 10.1093/geroni/igae098.1981

**Published:** 2024-12-31

**Authors:** Aaron Colverson

**Affiliations:** University of California San Francisco, San Francisco, California, United States

## Abstract

This presentation accounts for disciplinary similarities and differences between ethnomusicology and neuropsychology in the context of a project on rhythm perception, learning and performance, and cognitive functioning in healthy young and older adults. It draws from Judith Becker’s Deep Listeners (2004) and “Ethnomusicology and Empiricism in the 21st-century” (2009) to characterize many of the challenges present in building bridges between humanists and scientists studying music. Becker cites Ann Harrington in this regard, regarding what it might mean to teach an attitude of thinking neurobiologically about culture and ethnomusicologically about the brain. Applied to brain and social health in older adults, two notable scholars’ work extends from Becker’s and Harrington’s within the domain of ethnomusicology: Jennie Gubner and Theresa Allison. Building from these prior influences, my work forged a collaboration between neuropsychologists interested in the cognitive and brain health of older adults, with ethnomusicologists interested in contributing to new directions for the field, such as cognitive and medical ethnomusicologies. Through this collaboration, important questions were raised in the execution and evaluation phases of the project, namely: what counts as knowledge; what counts as evidence; how do we gather information; and how do we draw conclusions? By entering these conceptual domains/discussions between these disciplines, this work exemplifies a bridge toward a neuro-ethnomusicology of aging, prolonging the pioneering works of Becker, Harrington, Gubner, and Allison. The conclusions direct attention to more fluidly include both disciplines’ traditions in research design, to promote more collaborative work between humanists and scientists studying music and health.

